# Adams-Oliver syndrome: an unusual congenital disorder

**DOI:** 10.1093/omcr/omaf176

**Published:** 2025-09-28

**Authors:** Leen Jarjanazi, Sarah Kebbeh, Eymar Alam, Qamar Saado, Mulham Jarjanazi, Lama Alkadi, Aladdin Etr, Taoufik Ghazal Aswad, Hamdi Nawfal

**Affiliations:** Aleppo University Obstetrics and Gynecology Department- Faculty of Medicine, Aleppo, Syria; Aleppo University Obstetrics and Gynecology Department- Faculty of Medicine, Aleppo, Syria; Aleppo University Obstetrics and Gynecology Department- Faculty of Medicine, Aleppo, Syria; Aleppo University Obstetrics and Gynecology Department- Faculty of Medicine, Aleppo, Syria; Pediatric Surgery Department- Aleppo University Hospital, Aleppo, Syria; Aleppo University Hospital, Aleppo, Syria; Aleppo University Hospital, Aleppo, Syria; Aleppo University Hospital, Aleppo, Syria; Obstetrics and Gynecology - Embryology and Genetics Department- Aleppo University Faculty of Medicine, Aleppo, Syria

**Keywords:** Adams–Oliver syndrome, aplasia cutis congenita, brachysyndactyly, case report

## Abstract

Background: Adams-Oliver syndrome (AOS) is a rare congenital disorder characterized by scalp and limb malformations, including scalp aplasia and digital anomalies such as brachydactyly or oligodactyly. While typically inherited through either autosomal dominant or recessive patterns, sporadic cases have also been documented. Case Presentation: A male neonate, born to consanguineous parents, presented with classic features of AOS including aplasia cutis congenita (ACC) and terminal transverse limb defects (TTLD). The mother reported antidepressant use during the first trimester. Prenatal ultrasound findings were suggestive of AOS, which was confirmed postnatally by a large vertex scalp defect with absent skin and bone along with bilateral brachysyndactyly. All biochemical tests were normal, with no evidence of cardiovascular or neurological abnormalities. Conclusions: This case highlights the critical importance of early prenatal diagnosis for severe AOS through meticulous sagittal plane ultrasonography to detect vertex bone ossification defects. Given the poor postnatal prognosis, early recognition is essential to improve outcomes through timely intervention.

## Background

Adams-Oliver syndrome (AOS) is an uncommon disorder affecting approximately 1 in 225 000 births [1]. First identified by American clinical geneticist Forrest H. Adams and pediatric cardiologist Clarence Paul Oliver in a family with eight affected members [2], AOS presents with characteristic features including aplasia cutis congenita (a skin development abnormality) and limb malformations such as syndactyly, brachydactyly, or oligodactyly [3].

In some cases, incomplete ossification of the skull underlying the affected scalp area may occur, resulting in scarring and permanent alopecia [3]. The lower extremities are typically more severely affected than the upper limbs [4]. Additional manifestations may include cardiovascular anomalies (ventricular septal defects, Tetralogy of Fallot), vascular abnormalities, pulmonary hypertension, cutis marmorata telangiectatica congenita, and less frequently, hepatic, renal, or ocular involvement [1, 4]. Rare neurological findings have been reported, including cerebral cortical dysplasia, corpus callosum hypoplasia, ventricular dysmorphism, periventricular calcifications, and polymicrogyria [5–8].

Six genes have been associated with AOS, demonstrating either autosomal dominant or recessive inheritance patterns [9]. The causative genes include DLL4, ARHGAP31, and NOTCH1 (autosomal dominant) along with EOGT, DOCK6, and RBPJ (autosomal recessive) [9].

Treatment varies according to symptom severity and clinical presentation [10]. Systemic involvement, particularly of internal organs, significantly affects prognosis and may lead to fatal outcomes [11].

## Case presentation

**Figure 1:**
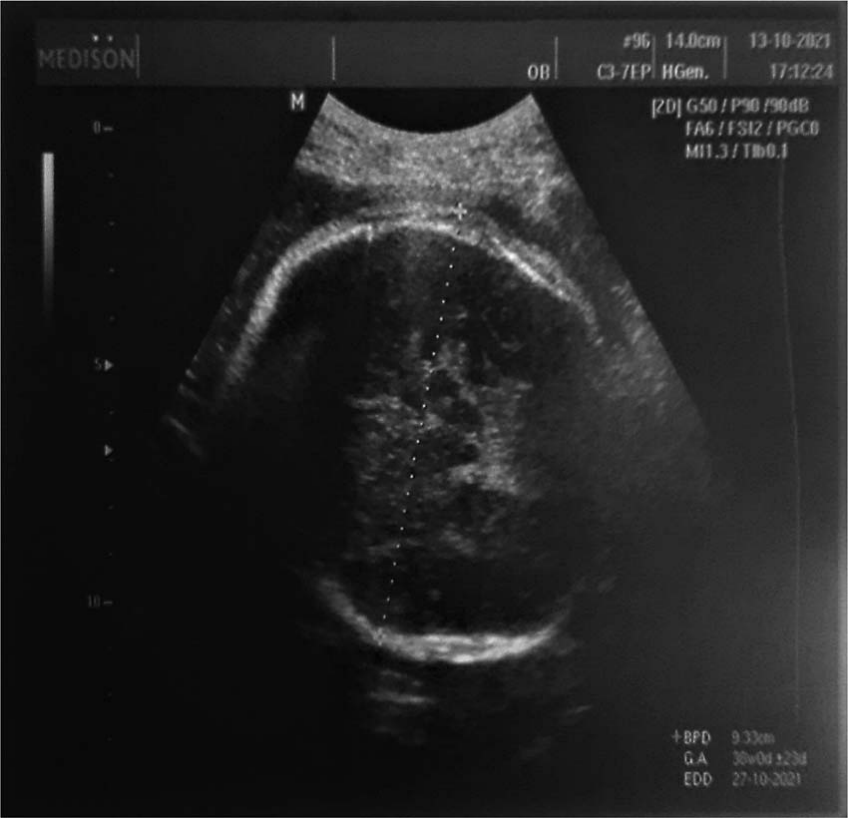
The figure shows an absence of ossification of the parietal area of the skull which indicates severe aplasia cutis congenita suggesting (AOS) syndrome as a differential diagnosis.

**Figure 2:**
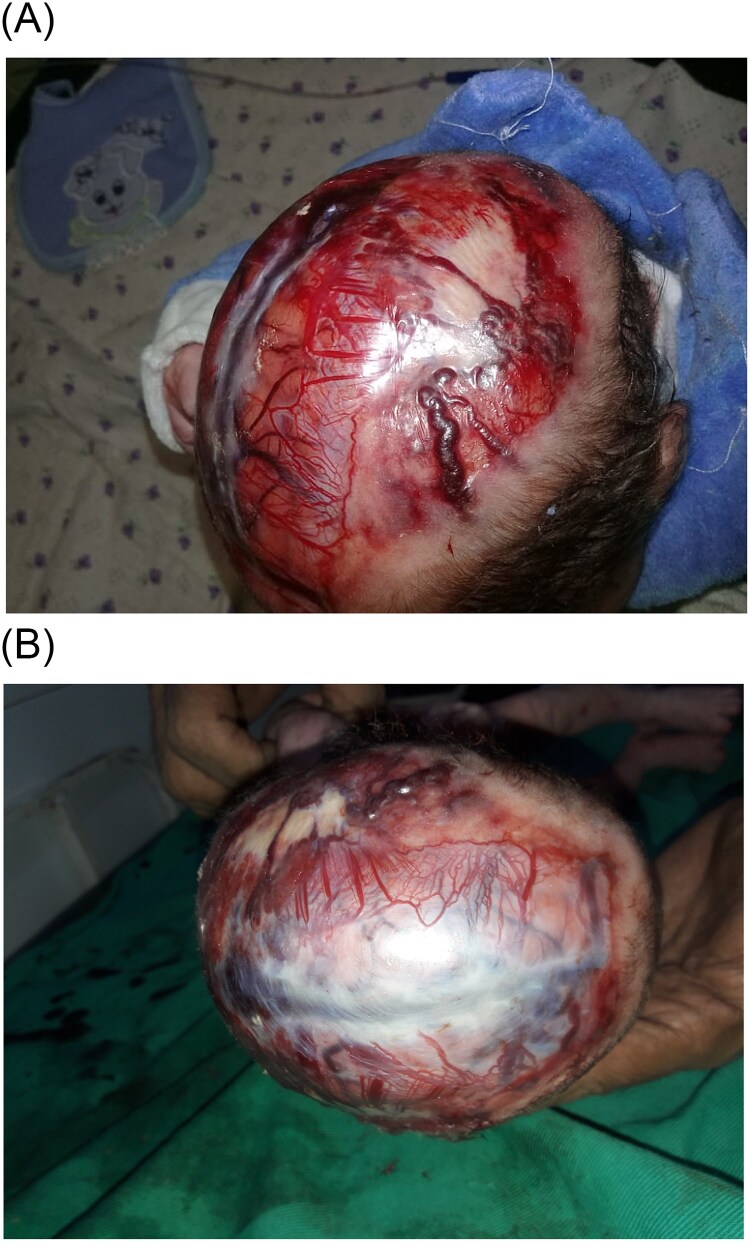
(A, B) an absence of the bone and the skin overlying the neonate’s scalp vertex which measures 11 × 9 cm. (A) Severe aplasia cutis congenita extending to the bone of the skull. (B) the superior sagittal venous sinus is clearly visible, which is a risk factor that may lead to severe bleeding or infection.

**Figure 3:**
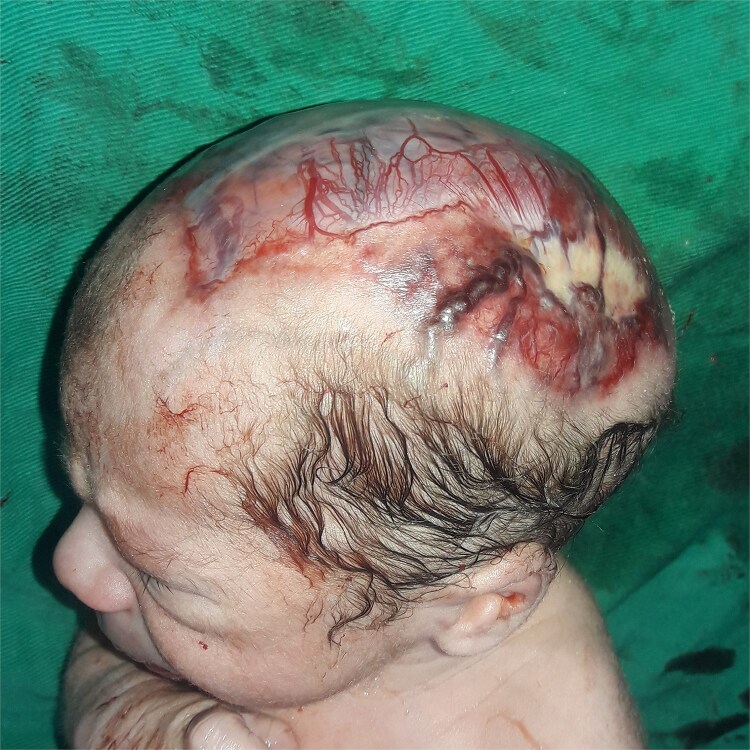
The appearance of the exposed meninges and dilated blood vessels.

**Figure 4:**
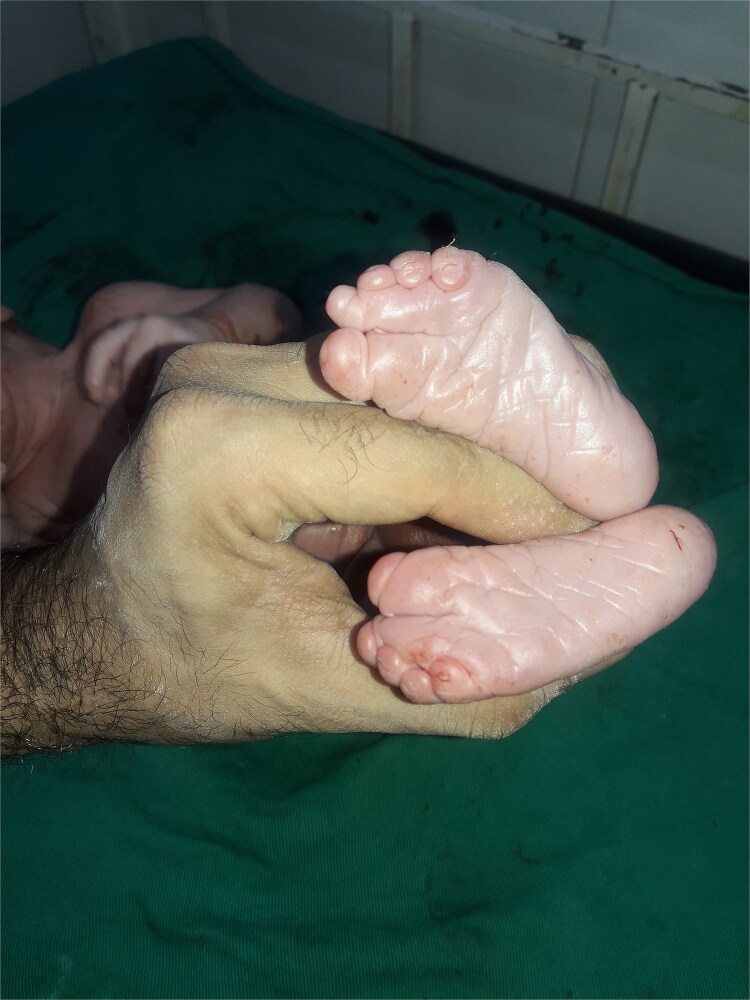
The figure shows shortening and fusion of the fingers in the bilateral lower limbs.

**Figure 5:**
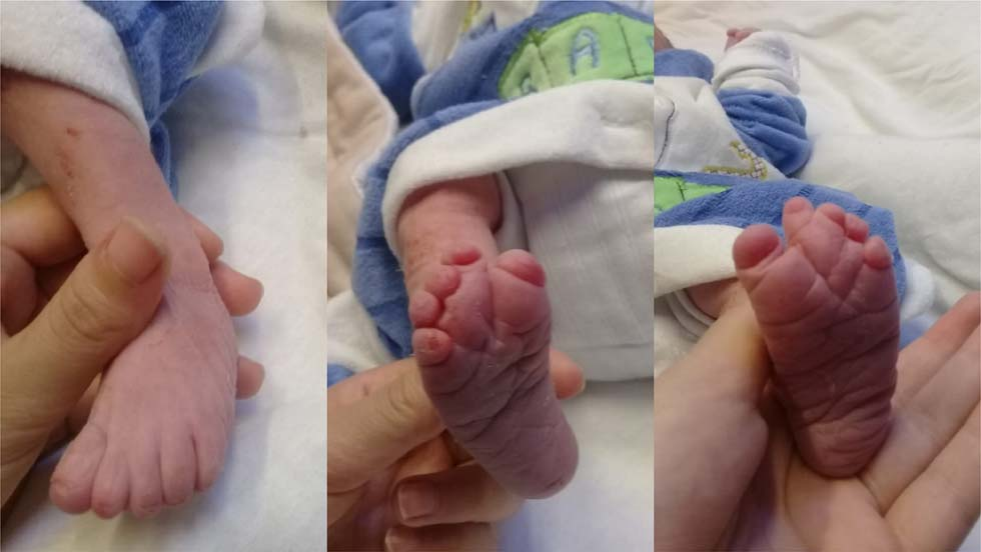
More figures showing the shortening and fusion of the fingers in the bilateral lower limbs.

An 18-year-old primigravida at full term presented to the Obstetrics and Gynecology Department with labor and fetal distress. An emergency cesarean section (EmCS) was performed, delivering a 2800 g newborn. The mother reported using nortriptyline (50 mg daily) during her first trimester. Prenatal ultrasound had demonstrated absent parietal bone ossification, initially misinterpreted as an imaging artifact. Due to this misinterpretation, a fetal echocardiography was not performed. [Fig. 1]. The mother’s medical history was otherwise unremarkable, though consanguinity was noted (her husband was her cousin). At delivery, the neonate presented with a large (11 × 9 cm) vertex scalp defect involving complete absence of skin and bone, exposing meninges and venous sinuses [Figs 2A,B and 3], along with bilateral lower extremity brachysyndactyly [Figs 4, 5]. Other physical and neurological examinations were normal, as were biochemical tests and abdominal ultrasonography. The neurosurgery and plastic surgery teams were consulted for management. Initial care included daily Fucidin gauze dressings to protect the defect, IV antibiotic therapy, and preparation for skin grafting. However, the lesion developed fibrous exudate with progressive recession. During preoperative preparation, localized infection progressed to bacteremia. The neonate’s condition rapidly deteriorated, culminating in death several days postpartum.

## Discussion

Adams-Oliver syndrome (AOS) is a rare disorder characterized by aplasia cutis congenita (ACC) and terminal transverse limb defects (TTLD) [4]. The diagnosis of AOS requires three key criteria: (1) terminal transverse limb abnormalities, (2) ACC, and (3) a positive family history [12]. Additional minor diagnostic features include vascular defects, congenital heart disorders, and cutis marmorata. A diagnosis can be established with either one major and one minor criterion or two major criteria [12].

A hallmark of AOS is its highly variable genotype–phenotype correlation, underscoring the need for a systematic and adaptable multidisciplinary approach in patient evaluation [13].

In our case, the infant presented with severe ACC involving the skull, exposing the meninges, along with TTLD limited to the lower extremities. Although no cardiovascular or neurological abnormalities were detected, the neonate succumbed to a secondary infection within days. Prenatal ultrasound revealed absent parietal skull ossification, a finding that could have supported an earlier AOS diagnosis; however, it was initially dismissed as an artifact.

While ultrasound is not universally reliable for diagnosing AOS—particularly in mild to moderate cases where ACC and TTLD may be subtle—it remains a critical tool in severe cases, such as ours. Early detection is vital, given the poor prognosis associated with severe AOS, and prenatal diagnosis may improve outcomes.

Further diagnostic measures, such as sagittal ultrasound, fetoscopy, or genetic testing (via amniocentesis or chorionic villus sampling), could enhance prenatal detection. Prenatal diagnosis not only provides pathogenic insights but also helps parents understand the severity of the condition.

The exact pathogenesis of AOS remains unclear [14]. Although a genetic component is suspected, our case showed no familial history of similar manifestations, despite consanguinity. This suggests a possible isolated occurrence, though genetic factors cannot be entirely excluded.

While antidepressant use in the first trimester raises concerns, no studies have established a link between antidepressants and AOS. Research indicates a potential association with cardiovascular malformations, but no significant increase in overall congenital malformation risk has been confirmed [15].

## Data Availability

All data generated or analysed during this study are included in this published article and its supplementary information files.

## Follow up

By the time we prepared this case report, the mother had become pregnant again. She discontinued all previous medications and received appropriate prenatal care, ultimately delivering a healthy baby boy.

## References

[1] Meester JAN, Southgate L, Stittrich A-B. et al. Heterozygous loss-of-function mutations in DLL4 cause Adams-Oliver syndrome. Am J Hum Genet 2015;97:475–82. 10.1016/j.ajhg.2015.07.015 26299364 PMC4564989

[2] Suarez E, Bertoli MJ, Eloy JD. et al. Case report and review of literature of a rare congenital disorder: Adams-Oliver syndrome. BMC Anesthesiol 2021;21:117. 10.1186/s12871-021-01339-0 33858352 PMC8048247

[3] Shiji Chalipat, Kiran CR, Mathew A, Mane S. Adams-Oliver Syndrome: Piecing Together the Puzzle of a Rare Condition. Medical Journal of Dr D Y Patil Vidyapeeth. 2025;18:526–528. 10.4103/mjdrdypu.mjdrdypu_1003_24

[4] Lehman A, Wuyts W, Patel MS. Adams-Oliver Syndrome. In: GeneReviews® [Internet]. Adam MP, Ardinger HH, Pagon RA. et al. eds. University of Washington, Seattle: Seattle (WA), 2016, 1993–2022.

[5] Pérez-García C, Martín YR, Del Hoyo AA. et al. Adams-Oliver syndrome with unusual central nervous system findings and an extrahepatic portosystemic shunt. Pediatr Rep 2017;9:7211. 10.4081/pr.2017.7211 28706620 PMC5494440

[6] Amor DJ, Leventer RJ, Hayllar S. et al. Polymicrogyria associated with scalp and limb defects: variant of Adams-Oliver syndrome. Am J Med Genet 2000;93:328–34. 10.1002/1096-8628(20000814)93:4<328::aid-ajmg13>3.0.co;2-0 10946361

[7] Savarirayan R, Thompson EM, Abbott KJ. et al. Cerebral cortical dysplasia and digital constriction rings in Adams-Oliver syndrome. Am J Med Genet 1999;86:15–9. 10.1002/(sici)1096-8628(19990903)86:1<15::aid-ajmg4>3.0.co;2-i 10440823

[8] Tan AP, Mankad K. Adams Oliver syndrome with cerebellar cortical dysplasia. Childs Nerv Syst 2018;34:1109–10. 10.1007/s00381-018-3810-1 29680918

[9] Zhu, Victor Z. et al. “Adams-Oliver Syndrome: Vestigial Tail and Genetics Update.” Archives of Plastic Surgery 2022;49:517–522. 10.1055/s-0042-1751107 PMC9340189 35919556

[10] Beekmans SJ, Wiebe MJ. “Surgical treatment of aplasia cutis in the Adams-Oliver syndrome.” The Journal of Craniofacial Surgery 2001;12:569–72. 10.1097/00001665-200111000-00014 11711825

[11] Srihari S, Asha GS. Adams-Oliver syndrome - a case report. Indian Dermatol Online J 2022;14:422–3. 10.4103/idoj.idoj_330_22 37266096 PMC10231716

[12] Rashid S, Azeem S, Riaz S. Adams-Oliver syndrome: a rare congenital disorder. Cureus. 2022;14:e23297. 10.7759/cureus.23297 35449659 PMC9012592

[13] Dudoignon B, Huber C, Michot C. et al. Expanding the phenotype in Adams–Oliver syndrome correlating with the genotype. Am J Med Genet A 2020;182:29–37. 10.1002/ajmg.a.61364 31654484

[14] Iftikhar N. Ahmad Ghumman FI, Janjua SA, Ejaz A, Butt UA. Adams-Oliver syndrome. J Coll Physicians Surg Pak. 2014;24:76–7. 24906278

[15] Grigoriadis S, VonderPorten EH, Mamisashvili L. et al. Antidepressant exposure during pregnancy and congenital malformations: is there an association? A systematic review and meta-analysis of the best evidence. The Journal of clinical psychiatry 2013;74:e293–308. 10.4088/JCP.12r07966 23656855

